# Correction: Evaluation of short-term hair follicle storage conditions for maintenance of RNA integrity

**DOI:** 10.1371/journal.pone.0347215

**Published:** 2026-04-14

**Authors:** Eilís E. Harkin, John A. Browne, Barbara A. Murphy

There are errors in the column header and legend for Table 2. RNA (ng/ul) should read as RNA (ng/15uL). Please see the correct Table 2 here.

**Table 2:** RNA quantity (ng/15uL), RNA quality (RIN) and qPCR Cycle Threshold (CT) values for samples stored in RNAlater at −20°C (Group A), 4°C (Group B) and 19°C (Group C) for one week prior to RNA isolation.

Sample Number	Horse Number	Sample storage temperature	RNA (ng/15uL)	RIN	CT value	CT value
					H3F3A	RPL19
1	1	−20°C	446.23	9.4	20.55	19.10
2	2	−20°C	608.88	9	22.61	19.41
3	3	−20°C	798.87	9.9	21.18	20.22
4	4	−20°C	211.31	9.6	21.42	20.53
5	1	4°C	432.85	10	20.67	19.71
6	2	4°C	150.42	9.8	20.31	19.94
7	3	4°C	430.10	9.7	20.68	19.17
8	4	4°C	81.78	9.4	20.85	19.59
9	1	19°C	248.82	9.5	21.57	19.77
10	2	19°C	448.19	9.7	20.52	20.38
11	3	19°C	979.98	8.2	20.99	19.49
12	4	19°C	226.17	9.6	20.26	19.61

## References

[1] Harkin EE, Browne JA, Murphy BA. Evaluation of short-term hair follicle storage conditions for maintenance of RNA integrity. PLoS One. 2024;19(5):e0294089. doi: 10.1371/journal.pone.0294089 38820307 PMC11142484

