# Supplementary material for: Evaluation of short-term hair follicle storage conditions for maintenance of RNA integrity
Source: PLoS One. 2024 May 31;19(5):e0294089. doi: 10.1371/journal.pone.0294089 (PMC11142484; doi:10.1371/journal.pone.0294089)
Supplement: Attachment — Submitted filename: R2_PONE-23-34914.pdf [file pone.0294089.s003.pdf]

Dear authors,

Thank you for the submitted review.

I can agree with your choice to do a repeated, one way ANOVA to test for the effect of storage. By doing so you consider the within-subject correlation in your data. But I believe my suggestion of using a two-factor model would also work.

With respect to the power I do not agree with the computations or the wording on line 330. When you do a post-hoc analysis you need to use the averages of your data. In that case the computation of delta is not appropriate. Delta is the max(mean)-min(mean) in the data. For RIN your means are 9.475, 9.25 and 9.725 so the delta is 0.475 and the RootMSE=0.48 which leads to a ratio of approx..= 1 . The power to detect differences of this size is only 17%.

Δ/σ= 9.7 (Max value observed in present study) – 7 (minimum reported value from literature) / 0.48 (SD) => Δ/σ= 5.6, the

However if you would argue that you wanted to test if RIN values would be too low for any treatment (e.g. RIN=7) then I could follow your reasoning but that is not a proper post hoc test. So you could rephrase that you wanted to detect very large differences for which n=4 is sufficient.

"The small sample size used in this study may have represented a limitation. However, a post-hoc examination of our results using the B12 Table for determining sample size for analysis of variance from Kutner et al, (2005) (28), indicated that our sample size of n=4 was sufficient for adequate statistical power.

The output in SAS that I obtained on your data for RIN

The GLMPOWER Procedure

Fixed Scenario Elements

Dependent Variable	RIN
Source	storage
Error Standard Deviation	0.48
Total Sample Size	12
Alpha	0.05
Test Degrees of Freedom	2
Error Degrees of Freedom	9

Computed Power

Power
0.171
