# Supplementary material for: Evaluation of short-term hair follicle storage conditions for maintenance of RNA integrity
Source: PLoS One. 2024 May 31;19(5):e0294089. doi: 10.1371/journal.pone.0294089 (PMC11142484; doi:10.1371/journal.pone.0294089)
Supplement: Attachment — Submitted filename: Rebuttal letter.docx [file pone.0294089.s001.docx]

Dear Editor,

Thank you for the opportunity to revise our manuscript for further consideration by PLoS ONE. Please see detailed responses below (in blue font) addressing the Journal requirements and Reviewer comments. All revisions to the text of the manuscript are highlighted in yellow in the document “Revised Manuscript with Track Changes”.

Sincerely,

Barbara Murphy

Journal Requirements:

When submitting your revision, we need you to address these additional requirements.

1. Please ensure that your manuscript meets PLOS ONE's style requirements, including those for file naming. The PLOS ONE style templates can be found at 
<https://journals.plos.org/plosone/s/file?id=wjVg/PLOSOne_formatting_sample_main_body.pdf> and 
<https://journals.plos.org/plosone/s/file?id=ba62/PLOSOne_formatting_sample_title_authors_affiliations.pdf>

The manuscript now meets the style requirements, including those for file naming.

2. In your Data Availability statement, you have not specified where the minimal data set underlying the results described in your manuscript can be found. PLOS defines a study's minimal data set as the underlying data used to reach the conclusions drawn in the manuscript and any additional data required to replicate the reported study findings in their entirety. All PLOS journals require that the minimal data set be made fully available. For more information about our data policy, please see <http://journals.plos.org/plosone/s/data-availability>.

The minimal data set underlying the results are included in the manuscript. All the data required to replicate the reported study findings in their entirety appear in the Tables.

"Upon re-submitting your revised manuscript, please upload your study’s minimal underlying data set as either Supporting Information files or to a stable, public repository and include the relevant URLs, DOIs, or accession numbers within your revised cover letter. For a list of acceptable repositories, please see <http://journals.plos.org/plosone/s/data-availability#loc-recommended-repositories>. Any potentially identifying patient information must be fully anonymized.

There is no additional data or supplemental information. All data is provided in the manuscript.

Important: If there are ethical or legal restrictions to sharing your data publicly, please explain these restrictions in detail. Please see our guidelines for more information on what we consider unacceptable restrictions to publicly sharing data: <http://journals.plos.org/plosone/s/data-availability#loc-unacceptable-data-access-restrictions>. Note that it is not acceptable for the authors to be the sole named individuals responsible for ensuring data access.

We will update your Data Availability statement to reflect the information you provide in your cover letter.

3. We note you have included a table to which you do not refer in the text of your manuscript. Please ensure that you refer to Table 1 in your text; if accepted, production will need this reference to link the reader to the Table.

Apologies for this omission. We now refer to Table 1 in the text.

4. Please review your reference list to ensure that it is complete and correct. If you have cited papers that have been retracted, please include the rationale for doing so in the manuscript text, or remove these references and replace them with relevant current references. Any changes to the reference list should be mentioned in the rebuttal letter that accompanies your revised manuscript. If you need to cite a retracted article, indicate the article’s retracted status in the References list and also include a citation and full reference for the retraction notice.

The reference list has been reviewed, edited, renumbered and any new references and/or changes highlighted in yellow.

Additional Editor Comments:

The concept of the present study seems interesting. In this work, the authors address an original topic and highlight valid challenges associated with sample collection in remote locations where researchers may not always be in a position to follow optimal transport and storage guidelines. However, the reviewers raised some concerns and I highly recommend the authors to address them precisely before I could deliver a decision on this work.

[Note: HTML markup is below. Please do not edit.]

Reviewers' comments:

Reviewer's Responses to Questions **Comments to the Author**

1. Is the manuscript technically sound, and do the data support the conclusions?

The manuscript must describe a technically sound piece of scientific research with data that supports the conclusions. Experiments must have been conducted rigorously, with appropriate controls, replication, and sample sizes. The conclusions must be drawn appropriately based on the data presented.

Reviewer #1: Yes

Reviewer #2: Partly

2. Has the statistical analysis been performed appropriately and rigorously?

Reviewer #1: Yes

Reviewer #2: No

3. Have the authors made all data underlying the findings in their manuscript fully available?

The [PLOS Data policy](http://www.plosone.org/static/policies.action#sharing) requires authors to make all data underlying the findings described in their manuscript fully available without restriction, with rare exception (please refer to the Data Availability Statement in the manuscript PDF file). The data should be provided as part of the manuscript or its supporting information, or deposited to a public repository. For example, in addition to summary statistics, the data points behind means, medians and variance measures should be available. If there are restrictions on publicly sharing data—e.g. participant privacy or use of data from a third party—those must be specified.

Reviewer #1: Yes

Reviewer #2: No

We are unclear why Reviewer 2 has responded ‘No’ here as all of the data has been provided in the manuscript.

4. Is the manuscript presented in an intelligible fashion and written in standard English?

PLOS ONE does not copyedit accepted manuscripts, so the language in submitted articles must be clear, correct, and unambiguous. Any typographical or grammatical errors should be corrected at revision, so please note any specific errors here.

Reviewer #1: Yes

Reviewer #2: Yes

5. Review Comments to the Author

Please use the space provided to explain your answers to the questions above. You may also include additional comments for the author, including concerns about dual publication, research ethics, or publication ethics. (Please upload your review as an attachment if it exceeds 20,000 characters)

Reviewer #1: The authors highlight valid challenges associated with sample collection in remote locations where researchers may not always be in a position to follow optimal transport and storage guidelines. The objective of the study is clearly outlined, the method well described, data appear appropriately analysed and conclusions are supported by the results. Some minor revisions will help to clarify certain aspects of the manuscript. Extra discussion points and recommendations for future research would lend further depth to the discussion section.

We thank the reviewer for their comments and hope that we have adequately addressed each in the sections below.

Abstract:
Line 65: Clarify if 3 or 4 horses were involved (see comments below).

Four horses were used in this study, this error has been rectified throughout the manuscript.

Introduction:
Please provide references of the 'limited information on acceptable short-term storage conditions' that do exist.

The appropriate citations have now been added to the end of this sentence. These are:

1. Bradley BJ, Pastorini J, Mundy NI. Successful retrieval of mRNA from hair follicles stored at room temperature: implications for studying gene expression in wild mammals. Molecular Ecology Notes. 2005;5(4):961-4.
2. Kim SJ, Dix DJ, Thompson KE, Murrell RN, Schmid JE, Gallagher JE, Rockett JC. Gene expression in head hair follicles plucked from men and women. Ann Clin Lab Sci. 2006 Spring;36(2):115-26.

Perhaps the authors could provide a thought on why information on this subject is limited if there is a need for more flexibility in short-term storage of samples.

There was perhaps a perception that hair follicles do not yield sufficient biological material for molecular analyses, such that more invasive tissue biopsies were favoured. In addition, improvements in RNA isolation methodologies and recent publications highlighting the successful use of this tissue for gene expression analysis has identified a need for more flexibility in short-term storage of samples so that it can be used for a wider range of species maintained in non-laboratory conditions. Furthermore, the manufacturers recommendations on the use of RNAlater suggests that RNA degradation may occur at room temperature after 3 days and this could have disincentivised researchers to risk valuable samples by delaying the time to freezer storage.

Materials and Methods:
Please clarify the number of horses used in the study. Three horses are mentioned in lines 65 and 147. Four horses are mentioned in line 146 and four samples per group are listed in Table 2.

We thank the reviewer for highlighting these inconsistencies. There were four horses used for samples in the study and the errors identified have been corrected.

Please elaborate on how the sufficient number of samples required for statistical comparison of groups was obtained.

4 replicates per storage condition were assessed. As the current study represented a novel evaluation of storage of samples from a non-human animal, population standard deviation or estimated effect of treatment could not be determined to carry out a standard power test for calculation of samples size for ANOVA. Instead to determine the number of biological replicates to use the limited available literature evaluating hair follicle storage in humans was first considered. In a relevant previously published study evaluating storage conditions of hair follicles in humans, samples from only two subjects were used and only two replicates per experimental treatments were assessed (Bradley et al., 2005). However, three biological replicates are generally advised in gene expression experiments (Rieu and Powers, 2009) and a more recent human hair follicle transcriptome profiling study used 4 subjects. It was therefore decided that samples from 4 animals maintained in identical conditions and collected at a single time point would be used. Previous published experiments conducted by the research team suggested there would be little variability expected in the biological material collected from each horse (Watts et al, 2011;Collery et al., 2022) such that 4 represented a suitable sample size for assessment of the sample storage conditions.

Reference: Rieu I, Powers SJ. Real-time quantitative RT-PCR: design, calculations, and statistics. Plant Cell. 2009 Apr;21(4):1031-3.

(Additional references cited in manuscript)

Line 146: Please abbreviate UCD prior to this mention or spell out the college name.

UCD has been changed to University College Dublin.

Please provide the time of year the samples were collected.

All samples were collected at a single time point on the same day in October. The month has now been added to the text.

Please clarify for the reader at this point which group is the one following to manufacturer's recommendation.

Group A followed the manufacturer’s recommended protocol. This has now been indicated in the text.

Please clarify how 4 and 19 degrees were maintained and measured over 7 days.

A refrigerator set at 4 degrees Celsius was used and the laboratory conditions for room temperature were determined by a room thermostat controlling room heating.

Line 182: Please explain why a modification to the manufacturer's instructions was made.

A modification was made to ensure the removal of any residual wash buffer. The word ‘buffer’ was omitted from ‘wash buffer’ and has been corrected in the text. This modification was made as an extra precaution and was based on the advice of the senior laboratory technician who had considerable experience with this kit.

Line 185: Please change µl to µL.

This change has been made.

Line 187: Please change uL to µL.

This change has been made.

Line 189: Please remove parentheses around RIN.

This change has been made.

Line 194: Should it be 20 µL instead of 20 ul (as in line 211)? Please amend if that is the case.

This change has been made.

Line 196: Please remove the full stop after '3.0 µL'.

This change has been made.

Results:

Line 237-39: Please remove the speculation on why sample 8 yielded lower quantity of RNA from the results section. This needs to be discussed more thoroughly in the discussion section.

This sentence has been removed.

Line 241: Please remove the sentence concerning the numerically higher result in group B. The differences between groups are highlighted in the previous sentence. As there was no statistically significant difference between groups the numerically higher value of one group is irrelevant.

This sentence has been removed.

Table 2: Please add group names to the table and/or table description.

Group names have been added to the Table legend.

Please include a column showing the horse associated with each sample.

A column has been added which shows the horse number associated with each sample.

It was speculated that sample 8 had thicker hair which may have affected results. If the animal in question had thicker hair than its samples may have had lower results in all groups. As there are a number of samples with lower RNA quantities it would be interesting to see if they were from the same animal.

In response to the reviewer’s query, we ran a one-way ANOVA on all parameters by horse. Whereas there was no significant effect of horse on RIN or CT values (p=0.86 and p=0.96, respectively), horse did effect the concentration of RNA obtainedand these samples were lower (p=0.0433). This suggests that the visibly observed greater hair thickness did impact the RNA isolation process. However, it is important to note that the lowest obtained RNA quantity (81.78ng/uL) still resulted in a total yield of 1.2ug of RNA (as 15uL was eluted). As the aim of the study was to compare the effectiveness of storage treatments, and no differences were observed statistically in this regard, we feel it suffices to address the lower concentration values related to hair thickness in the discussion.

Discussion section:
Line 288: Remove the word 'to' from 'before to freezing'.

This error has been corrected.

Line 312: Please elaborate on:

a) How thickness was assessed

Differences in thickness of hair shafts were visibly observed only.

b) How much thicker hairs in this sample were relative to those from other samples

This was not quantitatively assessed.

c) How thickness may have affected results

Thickness likely impacted the efficacy of the kit extraction columns. It is possible that the thicker hairs increased the viscosity of the homogenate following tissue lysis and led to a lower quantity of RNA.

d) Whether the 'thicker' hair from the animal that provided sample 8 also yielded lower RNA quantities in the other groups

Additional statistical analysis confirmed that samples from Horse 4 yielded lower mean RNA concentrations compared to samples from the other horses. As the aim of the study was to evaluate the impact of the different storage conditions on RNA concentration, quality and subsequent CT values during PCR validation, the authors respectfully suggest that it is not necessary to include the additional analysis in the manuscript. However, the impact of thicker hairs on extraction efficiency is highlighted and expanded upon further in the discussion in light of the reviewers comments.

The relevant section in the discussion has been revised as follows:

In the current study, lower concentrations of RNA were obtained in samples from Horse 4. Hairs from this horse appeared visibly thicker than the other sampled horses and it is likely that the larger diameter hair shafts may have increased the viscosity of the homogenate and compromised the performance of the RNA isolation kit extraction columns, reducing the concentration of RNA isolated. This highlights the importance of trimming the hairs to just below the follicles and taking care to reduce the number of hairs collected per sample in animals with particularly thick hairs.

Please discuss which cycle phase (anagen, catagen or telogen) samples may have been in at the time of sample collection.

As the horses in this study were all exposed to natural changes in environmental photoperiod, it is assumed that the hair coats were in the anagen phase of the hair growth cycle in October, in response to the shortening daylight hours that stimulates active growth of the heavier winter coat (O’Brien et al., 2020). As specific breed details for the study horses were unavailable, it is possible that Horse 4 was a more rustic breed. Breed rusticity is suggested to impact the response of the coat to environmental lighting changes such that at the time of sampling, this horse may have had a heavier winter coat and thicker hairs. These points has now been added to the discussion as follows:

As the horses in this study were all exposed to natural changes in environmental photoperiod throughout the year, it is assumed that their hair coats were in the anagen phase of the hair growth cycle at the sampling time in October, in response to the shortening daylight hours that stimulates active growth of the heavier winter coat (O’Brien et al., 2020). As specific breed details for the study horses were unavailable, it is possible that Horse 4 was a more rustic breed. Breed rusticity is suggested to impact the response of the coat to environmental lighting changes such that at the time of sampling, this horse may have had a heavier winter coat and thicker hairs. Despite this observation, the lowest RNA quantity obtained was >1.2 µg when accounting for the elution volume, which is surplus to the requirements of any gene expression assay. These results should help refine methodologies for future animal studies to improve the reliability, repeatability and quantities of RNA achieved

Could samples at a different phase have reacted differently to the different storage treatments?

There is no evidence to suggest that storage treatments would be impacted by the stage of the hair growth cycle. Introduction of follicle cells into RNAlater solution immediately freezes the transcriptome and this is effective with substantially denser tissue such as muscle. The phase of the cycle would however impact the metabolic activity of the hair follicle and the proportion of differentially expressed genes at time of sampling.

The authors mention that limited information on this subject has been published so far. Please discuss what future research may be needed to improve the current level of available information. For example:

a) In remote locations with higher ambient temperatures than 19 degrees

It would be interesting to evaluate the stability of RNA from samples collected and stored in RNAlater under higher short-term storage temperatures to emulate sample collections from animals in hotter climates. This could be carried out by storing the samples in incubators set at different temperatures and comparing the RNA quality and quantity obtained to samples maintained at the recommended conditions. This suggestion for a future study has been added to the discussion.

b) Should this study be repeated using hair follicles at different hair cycle phases

Respectfully, we do not feel this would be informative as apart from taking care to reduce the number of hair follicles collected when hairs are thicker, and trim the hairs as close to the follicle as possible, the hair cycle phases primarily impact the gene expression profile of the sample for specific hair cycle specific molecular pathways.

Reviewer #2: Dear authors,

The topic of your article is certainly interesting as it could provide useful information for sampling in the field.
I also believe that the collection and handling of samples and the extraction of RNA have been done properly.

Dear reviewer, thank you very much for your considered review of our manuscript. We hope that we have adequately addressed your comments below.

My main issue is the sample size that you have used and the subsequent conclusions that you draw from that.
With small sample size, it is quite probable to detect only large differences with reasonable power. Small differences might go undetected.

We do agree that it is probable to detect only large differences using smaller sample sizes. However, given that the RNA molecule can be affected by multiple degradation reactions and is known to be highly unstable unless sampled tissues are immediately snap frozen at -80 Celsius, or a stabilisation solution such as RNAlater is used as directed by the manufacturer, large differences in RNA quantity and quality would have been expected. Thus, smaller sample sizes would still provide sufficient power in studies such as this. We further expand on this below.

So my first question is why you decided on a sample size of 4. Could you report the power of the experiment so that it is clear that you had reasonable chance to detect differences (if they were there)?

Respectfully, for novel studies it is often not feasible to calculate power with any degree of accuracy, as population standard deviation or estimated effect of treatment cannot be determined to carry out a standard power test for calculation of sample size for ANOVA . Instead, to determine the number of biological replicates to use, we first reviewed the limited available literature evaluating hair follicle storage in humans. In a relevant previously published study evaluating storage conditions of hair follicles in humans, samples from only two subjects were used and only two replicates per experimental treatments were assessed (Bradley et al., 2005). However, three biological replicates are generally advised in gene expression experiments (Rieu and Powers, 2009) and a more recent human hair follicle transcriptome profiling study used 4 subjects (Zhang et al., 2017). It was therefore decided that samples from 4 animals maintained in identical conditions and collected at a single time point would be sufficient. Previous published experiments conducted by the research team suggested there would be little variability expected in the biological material collected from each horse (Watts et al, 2011;Collery et al., 2022) such that 4 represented a suitable sample size for assessment of the sample storage conditions.

Reference: Rieu I, Powers SJ. Real-time quantitative RT-PCR: design, calculations, and statistics. Plant Cell. 2009 Apr;21(4):1031-3.

(the remaining references cited here appear in the manuscript)

We also believe that the results support the suitability of the chosen sample size. Apart from the finding of no statistical difference between groups for any parameter, the numerical values of the results are equally relevant to the conclusions. Small differences in RIN value or RNA concentrations for example are not meaningful when such high mean values were achieved in the results. The minimum acceptable RIN value for an RNAseq analysis from samples derived from human hair was 7 (Zhang et al., 2017). All but one sample in the current study resulted in RNA with a RIN value less than 9.4 The average total yield of RNA in the current study was 6,321 ng (mean concentration/uL x 15 uL elution volume. To put this in perspective, Kim et al., (2006) in their study evaluating gene expression in human head hair follicles, yielded total RNA of <20 ng in half of their samples.

Secondly, from table 2, I cannot infer which horse delivered which samples. Because from the text, I understand that you sample 3 bunches of hair from each of the 4 horses . Is that correct?

Yes that is correct, and the omission of the Horse number in the Table was an oversight which has now been corrected.

If the sampling was done in that way, why not using 'horse' as the second factor in a model. That brings me to the second remark of doing a one-way ANOVA. With horse and treatment, you could do a two way ANOVA which could be more powerful.

Unfortunately, as there is only a single sample from each horse contributing to each treatment parameter, it is not possible to run a two-way ANOVA with the data. As this experiment aimed to evaluate storage conditions of a biological sample, rather than differences in the source of the biological samples, we do not feel that further analyses of the effects of horse are necessary.

And, did you check if assumptions for performing ANOVA were fulfilled (normally distributed residuals, homogeneous variance)?

Yes, the data passed the Shapiro-Wilks test for normality.

Finally, and this is more a conceptual remark, you are trying to prove equivalence of your treatment B and C to the standard method of A. This is different from trying to find a difference.

We agree, and believe the results do show equivalence of treatments to the standard method of A.

Text is written fluently and the article is concise.


L60. HF can be an easily accessible tissue but this sentence is very general. Not all gene expression in mammals can be interrogated! So this sentence should be rewritten.

You are correct, thank you. We have rephrased the sentence to read “Hair follicles provide an easily accessible tissue for interrogating gene expression for multiple purposes in mammals.”

L70 and following. to be reconsidered after re-analyzing.

We hope that the comments above have now satisfied your concerns in relation to the data analysis.

L147 three horses or 4?

Thank you, there were four horses and this typo has now been corrected.

L229 I do not really agree with the analysis. See comments above.

As mentioned above, a two-way ANOVA cannot be conducted when there is only a single sample from each horse contributing to each treatment group. A one-way ANOVA is the most appropriate analysis to compare means between groups for each parameter.
