# Supplementary material for: Evaluation of short-term hair follicle storage conditions for maintenance of RNA integrity
Source: PLoS One. 2024 May 31;19(5):e0294089. doi: 10.1371/journal.pone.0294089 (PMC11142484; doi:10.1371/journal.pone.0294089)
Supplement: Attachment — Submitted filename: Response to Reviewer 1.docx [file pone.0294089.s002.docx]

Rebuttal Letter

Dear Reviewer,

We thank you for your additional comments which we feel we have now addressed in full as detailed below.

Reviewer #2: Dear authors,


Thank you for reworking the article.

1) An explanation to the question of availability of the data&hellip;

Reviewer #1: Yes
Reviewer #2: No
We are unclear why Reviewer 2 has responded &lsquo;No&rsquo; here as all of the data has been
provided in the manuscript.
I initially choose for &ldquo;No&rdquo; because the horse ID was not provided. This is now corrected so you have provided all data.

Thank you.


2) I still miss answers to the statistical issues that I raised.

a) I disagree with you on the feasablity of a two-way anova. You have a perfectly crossed design. The 3 levels of factor &ldquo;storage&rdquo; are observed in all 4 horses (second factor). Horse can be considered as a blocking-factor.
So you could perfectly fit a 2 way model but assuming there is no interaction between storage*horse or you could use more elaborate models.

Some refs : Applied Linear Statistical Models, Chapter 20 by Kutner et al, https://users.stat.ufl.edu/~winner/sta4211/ALSM_5Ed_Kutner.pdf or
Alin A, Kurt S. Testing non-additivity (interaction) in two-way ANOVA tables with no replication. Statistical Methods in Medical Research. 2006;15(1):63-85. doi:10.1191/0962280206sm426oa

Respectfully, we have examined in-depth the reference provided by the reviewer and appreciate the points raised, but still do not agree that a two-way ANOVA is appropriate to use here. A two-way ANOVA is used when there are two factors that can contribute variability in the response variable. For example, had we wanted to examine the three storage treatments from samples collected from two groups of horses (e.g. sick versus healthy), or from samples collected from horses at different time points (e.g. morning versus evening), then a two-way ANOVA would be an appropriate model and an interaction between the factors could then be estimated. In the study by Alin and Kurt (2006) cited by the reviewer, two medical data sets are used as examples to explain methods to test interaction in two-way ANOVA tables with no replication. However, in one example three treatments are tested on two different types of rabbit heart (in normal and hypercholesterolemic) and in the second sample, three treatments are tested on three groups of rats. Both of these represent suitable data sets for two-way ANOVA analysis.

We strongly believe that the four horses in this study represent subjects or experimental units, and not a blocking factor. Samples were collected at the same time under the same conditions from each subject. In a completely randomised one-way ANOVA design, subjects would have been randomly assigned to each treatment and this would have required 12 horses to provide a sample size per treatment of n=4. Instead, to reduce inter-individual variability, each horse in this study contributed a sample to each treatment. Based on this, and through further evaluation of the suitability of our statistical method spurred by the reviewer’s points, we see that a repeated measures one-way ANOVA is more appropriate. This compares the mean differences between the three treatments and also accounts for inter-individual variation between subjects. We have re-run all of the statistical tests for each parameter using repeated measures one-way ANOVA. While P values have changed slightly, the overall finding of no difference between treatments for any parameter holds.

b) On the sample size and the power:
I agree that often for transcriptomics and gene expression a very small number of samples is used. But I guess that is often also because the cost of RNAsequencing is prohibitive.

My point is that you conclude from your experiment &ldquo;no significant effects between storage methods&rdquo;. That is true based on your data but it could be due to small sample size and/or low power of the experiment, which would only allow for large effects to be detected.

One option is that you compute the power of the Ftest on your data retrospectively and add this in the discussion. You might have had only sufficient power (80%) to detect differences of the size of 2.5*standard error, based on, for example, TABLE B.12 Table for Determining Sample Size for Analysis of Variance (fixed factor levels model Applied Linear Statistical Models, in <https://users.stat.ufl.edu/~winner/sta4211/ALSM_5Ed_Kutner.pdf>)

We thank the reviewer for suggesting we compute the power of the F test retrospectively. Based on a Post-hoc examination of our study findings for the RIN values, using the B12 Table from the Kutner et al, 2005, (Applied Linear Statistical Models Fifth edition) reference provided and assuming a=0.05, b=0.80, r=3 (conditions), Δ/σ= 9.7 (Max value observed in present study) – 7 (minimum reported value from literature) / 0.48 (SD) => Δ/σ= 5.6, the table indicates that our sample size of n=4 is sufficient for adequate power. We have now added to the discussion that we have retrospectively confirmed by using the cited reference table that the sample size for the study was adequate. We have also addedthe Kutner et al reference to the bibliography.

And considering your answer

“Small differences in RIN value or RNA concentrations for example are not meaningful when such high mean values were achieved in the results. The minimum acceptable RIN value for an RNAseq analysis from samples derived from human hair was 7 (Zhang et al., 2017). All but one sample in the current study resulted in RNA with a RIN value less than 9.4 The average total yield of RNA in the current study was 6,321 ng (mean concentration/uL x 15 uL elution volume. To put this in perspective, Kim et al., (2006) in their study evaluating gene expression in human head hair follicles, yielded total RNA of &lt;ng in half of their samples&rdquo;.

I do agree but why do would you do a statistical analysis (ANOVA) on the data if everything is acceptable?

Point noted, thank you.


c) Finally, and this is more a conceptual remark, you are trying to prove equivalence of your treatment B and C to the standard method of A. This is different from trying to find a difference.
.....You answer: &quot;We agree, and believe the results do show equivalence of treatments to the standard
method of A.&quot;

But you did not test equivalence statistically so you can not really conclude this from the analysis.

We agree, that was poor wording in our response. Failure to find a difference is not the same as establishing equivalence.
