# Supplementary material for: Evaluation of short-term hair follicle storage conditions for maintenance of RNA integrity
Source: PLoS One. 2024 May 31;19(5):e0294089. doi: 10.1371/journal.pone.0294089 (PMC11142484; doi:10.1371/journal.pone.0294089)
Supplement: Attachment — Submitted filename: Rebuttal letter v.3.docx [file pone.0294089.s004.docx]

Rebuttal letter

Reviewer #2: Dear authors,

Thank you for the submitted review.

I can agree with your choice to do a repeated, one way ANOVA to test for the effect of storage. By doing so you consider the within-subject correlation in your data. But I believe my suggestion of using a two-factor model would also work.

We are glad that you are in agreement with our choice, thank you.

With respect to the power I do not agree with the computations or the wording on line 330. When you do a post-hoc analysis you need to use the averages of your data. In that case the computation of delta is not appropriate. Delta is the max(mean)-min(mean) in the data. For RIN your means are 9.475, 9.25 and 9.725 so the delta is 0.475 and the RootMSE=0.48 which leads to a ratio of approx..= 1 . The power to detect differences of this size is only 17%.
Δ/σ= 9.7 (Max value observed in present study) – 7 (minimum reported
value from literature) / 0.48 (SD) => Δ/σ= 5.6, the
However if you would argue that you wanted to test if RIN values would be too low for any treatment (e.g. RIN=7) then I could follow your reasoning but that is not a proper post hoc test. So you could rephrase that you wanted to detect very large differences for which n=4 is sufficient.

Thank you for this clarification. We have expanded this section and rephrased the wording on line 330 to state that we were interested in detecting large differences only, for which n=4 was considered sufficient. We have supported this by highlighting samples sizes used from similar previously published papers and referred to the differences between our results and the minimum accepted RIN value from the literature. We have removed the Kutner reference again. The paragraph now reads as follows:

The small sample size used in this study may have represented a limitation. In a relevant previously published study evaluating storage conditions of hair follicles in humans, samples from only two subjects were used and only two replicates per experimental treatments were assessed (Bradley et al., 2005). However, three biological replicates are generally advised in gene expression experiments (Rieu and Powers, 2009) and a more recent human hair follicle transcriptome profiling study used 4 subjects (Zhang et al. 2017). It was therefore decided that samples from 4 animals maintained in identical conditions and collected at a single time point would be sufficient. Previous published experiments conducted by the research team suggested there would be little variability expected in the biological material collected from each horse (Watts et al, 2011;Collery et al., 2022) such that 4 represented a suitable sample size for assessment of the sample storage conditions where we were interested only in detecting large differences in relation to RIN values. The minimum acceptable RIN value for an RNAseq analysis from samples derived from human hair was 7 (Zhang et al., 2017). All but one sample in the current study resulted in RNA with a RIN value less than 9.4.
